# Supplementary material for: Evaluation of the Metabolite Profile of Fish Oil Omega-3 Fatty Acids (n-3 FAs) in Micellar and Enteric-Coated Forms—A Randomized, Cross-Over Human Study
Source: Metabolites. 2024 May 7;14(5):265. doi: 10.3390/metabo14050265 (PMC11123365; doi:10.3390/metabo14050265)
Supplement: Supplementary file 1 [file metabolites-14-00265-s001.zip › metabolites-2997737-supplementary.pdf]

Evaluation of the metabolite profile of Fish Oil Omega-3 fatty acids (n-3 FAs) in Micellar and Enteric Coated Forms—A Randomized, Cross-Over Human Study: Supplementary Data

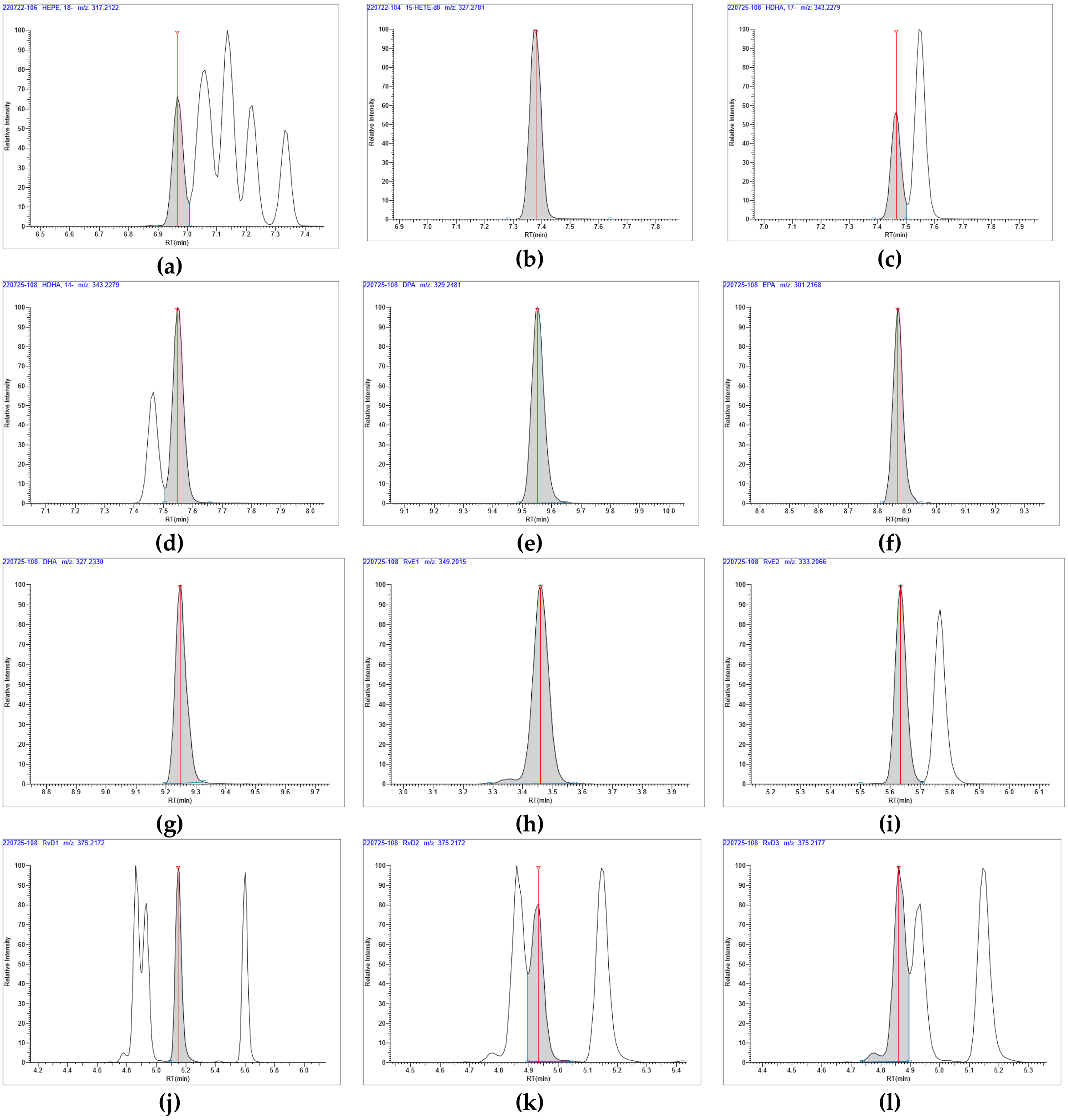

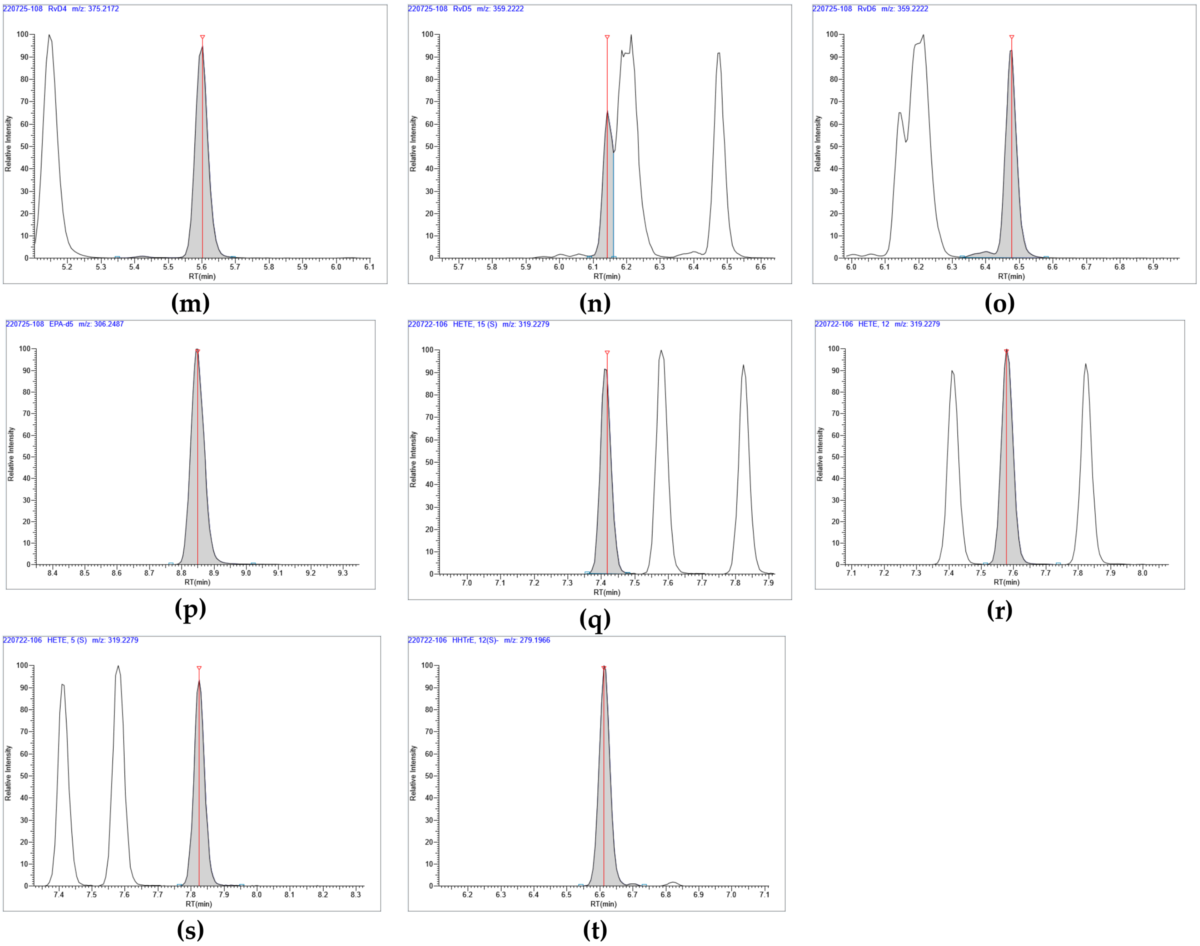

Figure S1: LC-HRMS chromatograms of n3-FAs and their metabolites. (a) 18-HEPE, (b) 15-HETE-d8, (c) 17-HDHA, (d) 14-HDHA, (e) DPA, (f) EPA, (g) DHA, (h) RvE1, (i) RvE2, (j) RvD1, (k) RvD2, (l) RvD3, (m) RvD4, (n) RvD5, (o) RvD6, (p) EPA-d5, (q) 15(S)-HETE, (r) 12(S)-HETE, (s) 5(S)-HETE, (t) 12(S)-HHTrE

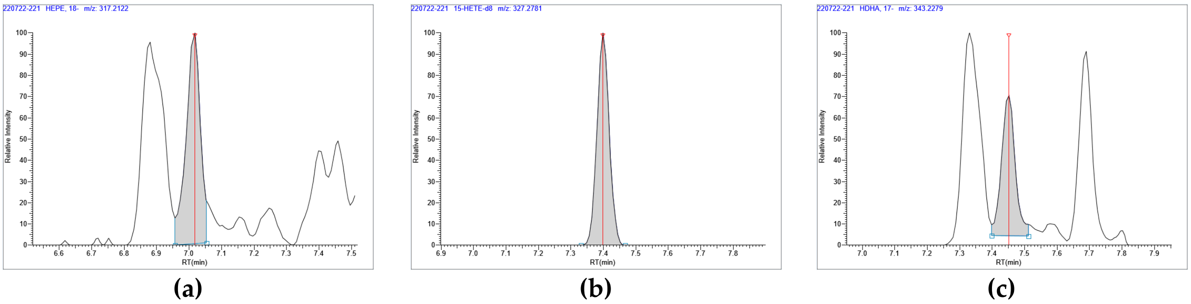

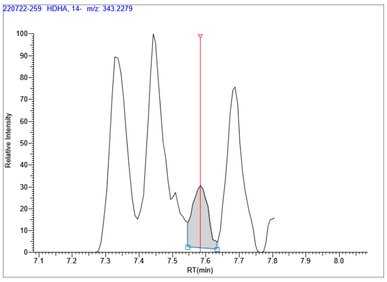

(d)

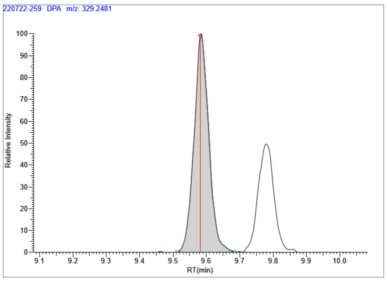

(e)

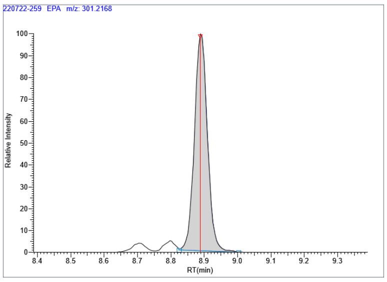

(f)

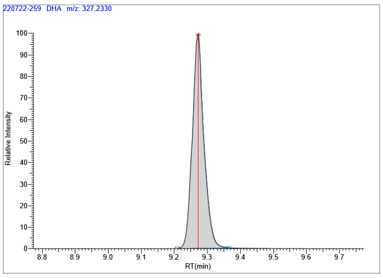

(g)

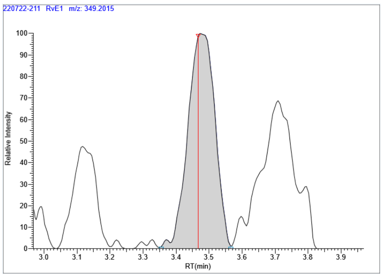

(h)

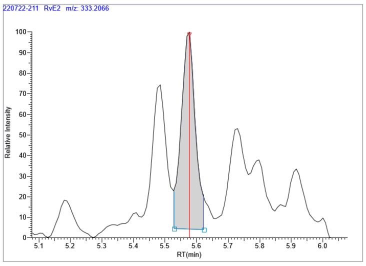

(i)

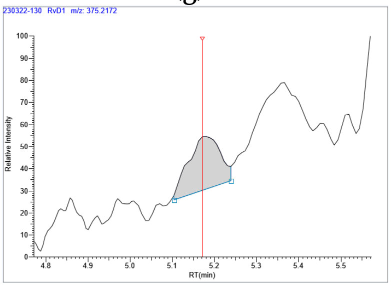

(j)

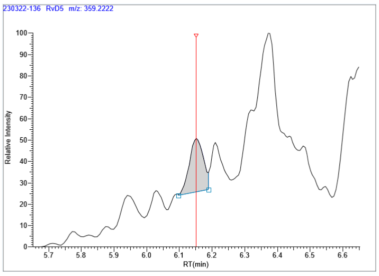

(k)

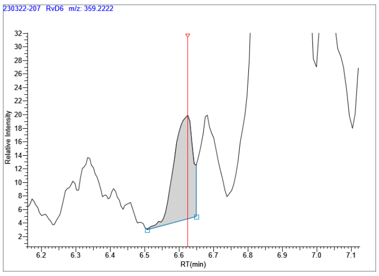

(l)

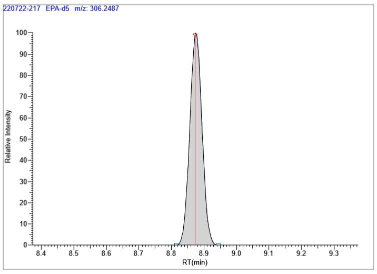

(m)

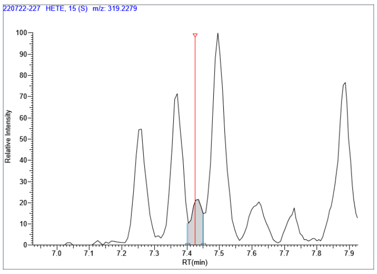

(n)

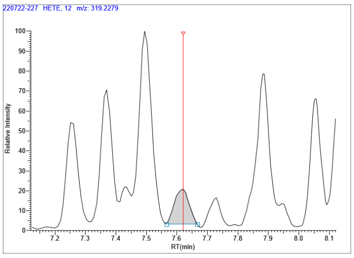

(o)

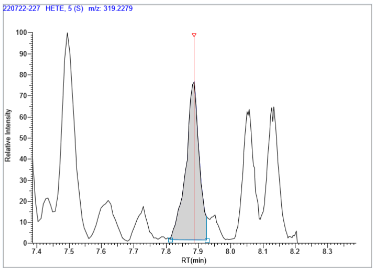

(p)

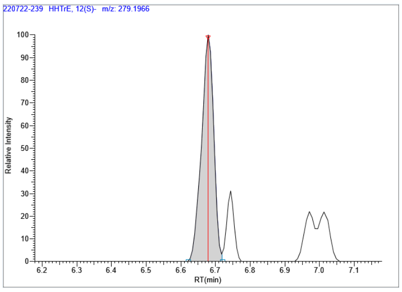

(q)

Figure S2: LC-HRMS chromatograms of n3-FAs and their metabolites as detected in participants' blood samples. (a) 18-HEPE, (b) 15-HETE-d8, (c) 17-HDHA, (d) 14-HDHA, (e) DPA, (f) EPA, (g) DHA, (h) RvE1,

(i) RvE2, (j) RvD1, (k) RvD5, (l) RvD6, (m) EPA-d5, (n) 15(S)-HETE, (o) 12(S)-HETE, (p) 5(S)-HETE, (q) 12(S)-HHTrE.

Table S1. HRMS data extraction parameters for n-3 FA and their metabolites

**Compound Name**	**Compound Formula**	**Adduct**	**Retention Time**
10-HDHA	C22H32O3	M-H	7.53
11-HDHA	C22H32O3	M-H	7.60
11-HEPE	C20H30O3	M-H	7.09
12(S)-HEPE	C20H30O3	M-H	7.25
12(S)-HETE	C20H32O3	M-H	7.60
12(S)-HHTrE	C17H28O3	M-H	6.66
14(s)-HDHA	C22H32O3	M-H	7.58
15(s)-HEPE	C20H30O3	M-H	7.10
15(S)-HETE	C20H32O3	M-H	7.26
15-HETE-d8	C20H24D8O3	M-H	7.26
17(S)-HDHA	C22H32O3	M-H	7.32
18-HEPE	C20H30O3	M-H	6.83
5(S)-HEPE	C20H30O3	M-H	7.33
5(S)-HETE	C20H32O3	M-H	7.85
6-keto_Prostaglandin_F1	C20H34O6	M-H	6.15
8-HEPE	C20H30O3	M-H	7.06
9-HEPE	C20H30O3	M-H	7.13
DHA	C22H32O2	M-H	9.12
DHA-d5	C22H27O2D5	M-H	9.12
DPA3	C22H34O2	M-H	9.41
EPA	C20H30O2	M-H	8.74
EPA-d5	C20H25D5O2	M-H	8.74
Mr1	C22H32O4	M-H	6.22
Mr2	C22H32O4	M-H	6.24
PrD1	C22H32O4	M-H	6.24
Prostaglandin E3	C20H30O5	M-H	4.36
Prostaglandin F3a	C20H30O5	M-H	4.36
Prostaglandin_D2	C20H32O5	M-H	4.87
Prostaglandin_E2	C20H32O5	M-H	4.78
Prostaglandin_F2a	C20H34O5	M-H	5.03
RvD1	C22H32O5	M-H	4.75
RvD2	C22H32O5	M-H	4.97
RvD3	C22H32O5	M-H	4.87
RvD4	C22H32O5	M-H	5.62
RvD5	C22H32O4	M-H	6.15
RvD6	C22H32O4	M-H	6.51
RvE1	C20H30O5	M-H	3.46

RvE2	$C_{20}H_{30}O_4$	M-H	5.65
Thromboxane_B2	$C_{20}H_{34}O_6$	M-H	4.51

Table S2: Serum triglycerides at various time points during treatment.

Time (h)	LMF	STD	ENT
0	2.14 ± 0.73	2.16 ± 0.68	1.27 ± 0.21
3	1.98 ± 0.50	2.24 ± 0.57	1.51 ± 0.24
6	2.34 ± 0.40	2.34 ± 0.53	1.78 ± 0.30
12	2.53 ± 0.67	2.58 ± 0.70	2.38 ± 0.49*
24	1.81 ± 0.52	2.09 ± 0.69	1.45 ± 0.33
48	1.49 ± 0.40*	2.99 ± 1.20*	1.35 ± 0.32
72	1.41 ± 0.24*	1.54 ± 0.38	1.44 ± 0.34
96	1.45 ± 0.29*	1.81 ± 0.46	1.24 ± 0.24
120	1.84 ± 0.61*	1.40 ± 0.37	1.38 ± 0.45

2-way ANOVA Repeated Measures using Time 0 as Control with Dunnet multiple comparison correction. * denotes $p < 0.05$.

Table S3: Blood HDL at various time points during treatment.

Time (h)	LMF	STD	ENT
0	3.80 ± 0.41	4.31 ± 0.61	4.10 ± 0.52
3	3.65 ± 0.34	4.38 ± 0.67	4.03 ± 0.57
6	3.78 ± 0.38	4.28 ± 0.69	4.28 ± 0.61
12	3.89 ± 0.47	4.04 ± 0.64	4.04 ± 0.68
24	3.88 ± 0.41	4.23 ± 0.68	4.32 ± 0.60
48	3.73 ± 0.40	4.19 ± 0.83	4.31 ± 0.56
72	3.78 ± 0.38	4.12 ± 0.66	4.10 ± 0.51
96	3.44 ± 0.39	4.09 ± 0.66	3.79 ± 0.42
120	3.63 ± 0.54	4.18 ± 0.66	4.17 ± 0.53

2-way ANOVA Repeated Measures using Time 0 as Control with Dunnet multiple comparison correction. No significance was found.

Table S4: Blood LDL at various time points during treatment.

Time (h)	LMF	STD	ENT
0	2.90 ± 0.4	3.31 ± 0.49	3.52 ± 0.47
3	2.78 ± 0.35	3.36 ± 0.49	3.34 ± 0.50
6	2.77 ± 0.36	3.21 ± 0.52	3.46 ± 0.52
12	2.81 ± 0.49	2.64 ± 0.40	3.09 ± 0.49
24	3.13 ± 0.47	3.28 ± 0.46	3.65 ± 0.49
48	3.05 ± 0.36	3.32 ± 0.65	3.69 ± 0.47
72	3.14 ± 0.37	3.94 ± 0.33	3.44 ± 0.39

96	2.78 ± 0.36	3.26 ± 0.49	3.22 ± 0.34
120	2.79 ± 0.49	4.04 ± 0.42	3.54 ± 0.41

2-way ANOVA Repeated Measures using Time 0 as Control with Dunnet multiple comparison correction. No significance was found.

Table S5: Blood LDL/HDL Ratio at various time points during treatment.

Time (h)	LMF	STD	ENT
0	2.03 ± 0.32	2.72 ± 0.54	2.23 ± 0.50
3	2.02 ± 0.29	2.94 ± 0.65	2.13 ± 0.45
6	2.24 ± 0.34	3.08 ± 0.79	2.51 ± 0.54
12	2.10 ± 0.39	2.43 ± 0.59	2.35 ± 0.57
24	2.27 ± 0.33	3.04 ± 0.64	2.33 ± 0.44
48	2.59 ± 0.41	2.93 ± 0.79	2.36 ± 0.44
72	2.46 ± 0.30	3.58 ± 0.23	2.27 ± 0.40
96	2.28 ± 0.40	2.78 ± 0.55	2.21 ± 0.42
120	2.54 ± 0.61	3.95 ± 0.32	2.65 ± 0.40

2-way ANOVA Repeated Measures using Time 0 as Control with Dunnet multiple comparison correction. No significance was found.

Table S6: Blood TC at various time points during treatment.

Time (h)	LMF	STD	ENT
0	5.2 ± 0.36	5.7 ± 0.38	5.88 ± 0.43
3	5.02 ± 0.35	5.73 ± 0.47	5.79 ± 0.5
6	5.03 ± 0.39	5.54 ± 0.47	5.87 ± 0.53
12	5.2 ± 0.42	5.23 ± 0.46	5.34 ± 0.52
24	5.26 ± 0.42	5.5 ± 0.5	6.03 ± 0.52
48	5.03 ± 0.35	5.81 ± 0.52	6.03 ± 0.49
72	5.15 ± 0.4	5.42 ± 0.51	5.77 ± 0.48
96	4.77 ± 0.34	5.44 ± 0.5	5.43 ± 0.33
120	4.92 ± 0.38	5.43 ± 0.49	5.59 ± 0.55

2-way ANOVA Repeated Measures using Time 0 as Control with Dunnet multiple comparison correction. No significance was found.

Table S7: Blood non-HDL cholesterol at various time points during treatment.

Time (h)	LMF	STD	ENT
0	3.8 ± 0.41	4.31 ± 0.61	4.1 ± 0.52
3	3.65 ± 0.34	4.38 ± 0.67	4.03 ± 0.57
6	3.78 ± 0.38	4.28 ± 0.69	4.28 ± 0.61
12	3.89 ± 0.47	4.04 ± 0.64	4.04 ± 0.68
24	3.88 ± 0.41	4.23 ± 0.68	4.32 ± 0.6
48	3.73 ± 0.4	4.19 ± 0.83	4.31 ± 0.56
72	3.78 ± 0.38	4.12 ± 0.66	4.1 ± 0.51

96	3.44 ± 0.39	4.09 ± 0.66	3.79 ± 0.42
120	3.63 ± 0.54	4.18 ± 0.66	4.17 ± 0.53

2-way ANOVA Repeated Measures using Time 0 as Control with Dunnet multiple comparison correction. No significance was found.

Table S8: Serum lipid parameters combining all treatments.

Time (h)	HDL	LDL	LDL/HDL	TC	TRG	non-HDL
0	1.55 ± 0.11	3.27 ± 0.26	2.29 ± 0.27	5.60 ± 0.23	1.80 ± 0.32	4.05 ± 0.28
3	1.52 ± 0.11	3.17 ± 0.26	2.31 ± 0.27	5.50 ± 0.26	1.86 ± 0.24	3.98 ± 0.3
6	1.38 ± 0.10*	3.14 ± 0.27	2.56 ± 0.31	5.45 ± 0.27	2.14 ± 0.23	4.08 ± 0.3
12	1.34 ± 0.10*	2.88 ± 0.26*	2.29 ± 0.29	5.26 ± 0.26	2.49 ± 0.33*	3.98 ± 0.32
24	1.47 ± 0.10*	3.36 ± 0.27	2.49 ± 0.26	5.59 ± 0.27	1.75 ± 0.28	4.12 ± 0.3
48	1.48 ± 0.11	3.36 ± 0.26	2.57 ± 0.28	5.60 ± 0.27	1.83 ± 0.38	4.06 ± 0.31
72	1.46 ± 0.10	3.42 ± 0.22	2.63 ± 0.22	5.43 ± 0.26	1.45 ± 0.17	3.98 ± 0.27
96	1.46 ± 0.11	3.09 ± 0.22	2.39 ± 0.25	5.21 ± 0.22*	1.47 ± 0.18	3.76 ± 0.27
120	1.33 ± 0.12*	3.42 ± 0.27	2.96 ± 0.30*	5.33 ± 0.27	1.53 ± 0.27	4.00 ± 0.31

2-way ANOVA Repeated Measures using Time 0 as Control with Dunnet multiple comparison correction. * denotes $p < 0.05$.
